# Protein kinase A-mediated phosphorylation of RhoA on serine 188 triggers the rapid induction of a neuroendocrine-like phenotype in prostate cancer epithelial cells

**DOI:** 10.1016/j.cellsig.2012.03.018

**Published:** 2012-08

**Authors:** Sarah E. Jones, Timothy M. Palmer

**Affiliations:** Institute of Cardiovascular and Medical Sciences, College of Medical, Veterinary and Life Sciences, University of Glasgow, Glasgow G12 8QQ, Scotland, UK

**Keywords:** PCa, prostate cancer, cAMP, cyclic AMP, NE, neuroendocrine, PSA, prostate-specific antigen, PKA, cAMP-dependent protein kinase, bFGF, basic fibroblast growth factor, NGF, nerve growth factor, 6-Bnz-cAMP, N^6^-benzoyl-cAMP, Fsk, forskolin, C3T, cell-permeable *C. botulinum* C3 transferase, CREB, cAMP response element binding protein, ERK, extracellular signal-regulated kinase, RACK1, receptor of activated C kinase 1, EPAC, exchange protein directly activated by cAMP, ROCK, Rho-associated protein kinase, Cyclic AMP, RhoA, Prostate cancer, Epithelial cells

## Abstract

Whilst androgen ablation therapy is used to treat locally advanced or metastatic forms of prostate cancer, side-effects can include the emergence of an androgen-independent neuroendocrine cell population which is associated with poor prognosis. Here we have examined how cyclic AMP elevation regulates early events in the neuroendocrine differentiation process. We demonstrate that selective activation of protein kinase A is necessary and sufficient for cyclic AMP (cAMP) elevation to rapidly promote a neuroendocrine phenotype in LNCaP cells independent of de novo protein synthesis. Furthermore, the effects of cAMP could be recapitulated by inhibition of RhoA signalling or pharmacological inhibition of Rho kinase. Conversely, expression of constitutively active Gln63Leu-mutated RhoA acted as a dominant-negative inhibitor of cAMP-mediated NE phenotype formation. Consistent with these observations, cAMP elevation triggered the PKA-dependent phosphorylation of RhoA on serine 188, and a non-phosphorylatable Ser188Ala RhoA mutant functioned as a dominant-negative inhibitor of cAMP-mediated neuroendocrine phenotype formation. These results suggest that PKA-mediated inhibition of RhoA via its phosphorylation on serine 188 and the subsequent inhibition of ROCK activity plays a key role in determining initial changes in cellular morphology during LNCaP cell differentiation to a neuroendocrine phenotype. It also raises the possibility that targeted suppression of this pathway to inhibit neuroendocrine cell expansion might be a useful adjuvant to conventional prostate cancer therapy.

## Highlights

► Cyclic AMP rapidly initiates a neuroendocrine phenotype in prostate cells. ► PKA is necessary and sufficient for initiation of the neuroendocrine phenotype. ► Inhibition of PKA-mediated phosphorylation of RhoA on Ser188 blocks the effect.

## 1 Introduction

Prostate cancer (PCa^I^) is the most frequently diagnosed male-specific malignancy and causes the greatest number of male cancer-related deaths after lung cancer [1]. Prostate cell growth is reliant on the presence of androgens such as testosterone with traditional therapeutic strategies focussing on androgen ablation therapy [1]. However, such therapies have unwanted side effects including mood alterations, sexual dysfunction, skeletal complications and increased risk of diabetes and cardiovascular events [2,3], and selection for the emergence of androgen-independent cells such as neuroendocrine (NE) cells [1]. NE cells play a key role in governing differentiation and proliferation of the developing prostate gland and, whilst their role in the mature gland is less well defined, it is thought that they play important regulatory roles in secretion [4].

Clinically, PCa incidence and progression are assessed via monitoring serum prostate specific antigen (PSA) levels. However PSA is limited as a biomarker due to issues with false-positive results and lack of discrimination between aggressive and non-aggressive diseases [5]. Thus other markers are being assessed for use in the diagnosis and prognosis of PCa. Recently, the presence of NE cell products as a prognostic marker in PCa has come under investigation [6]. The presence of NE cells has been described in 47 to 100% of prostatic adenocarcinomas [7]. Expansion of the NE cell population has been described in late-stage PCa and has been suggested to be indicative of poor patient prognosis [6]. NE cells form foci of non-dividing cells surrounded by regions of proliferating tumour cells due to the release of mitogenic compounds such as bombesin [8] and chromogranin A [9]. Due to their resistance to many conventional chemotherapeutics, it is possible that inhibition of NE cell differentiation may be a useful avenue by which to complement existing therapies and thus prevent expansion of this cell type in late-stage PCa.

The LNCaP cell line was isolated from a lymph node metastasis of PCa [10] and is a well-established model of NE differentiation in PCa. LNCaP cells undergo differentiation to a NE phenotype under a number of conditions including androgen deprivation [11], chronic exposure to IL-6 [12], constitutive activation of gp130 [13] and elevation of intracellular cyclic AMP (cAMP) levels [7,12,14]. Initially, the differentiation of these cells to a NE phenotype is reversible [15] but chronic exposure to cAMP-elevating agents ultimately results in the terminal differentiation of LNCaP cells [7]. Inhibition of the initial stages of NE differentiation in prostate epithelial cells may therefore be a useful adjuvant to conventional chemotherapeutic strategies. Of particular interest is the role of cAMP-mediated signalling in this phenomenon and elucidation of the signalling mechanisms involved in initial NE differentiation.

Classically, cAMP was thought to exert its intracellular effects exclusively via activation of cAMP-dependent protein kinase (PKA). Binding of cAMP to the PKA holoenzyme promotes dissociation of the regulatory and catalytic subunits [16,17] and allows the catalytic subunits of PKA to phosphorylate downstream targets, such as the cAMP responsive element binding protein (CREB) [18–20]. Phosphorylation of transcription factors such as CREB is one mechanism by which elevation of intracellular cAMP concentrations can alter gene transcription. However it is now appreciated that in addition to PKA, cAMP can activate the exchange proteins directly activated by cAMP (EPACs) to promote intracellular effects [21–23]. It has been previously demonstrated that over-expression of constitutively active PKA catalytic subunits in LNCaP cells can promote NE differentiation in the absence of other stimuli [14], suggesting that PKA activation is sufficient to recapitulate this phenomenon following cAMP elevation. Furthermore, PKA-differentiated LNCaP cells can promote the anchorage-dependent and -independent growth of prostate cells [24], indicating that the kinase may play a crucial role in PCa progression. The ability of cAMP elevation to promote a neuronal cell phenotype is not restricted to LNCaP cells. In the PC12 pheochromocytoma cell line, elevation of cAMP can promote neurite outgrowth [25], Unlike LNCaP cells in which differentiation can be induced through constitutive PKA activity, PC12 cells require signalling through EPAC for neuronal differentiation [26]. Clearly different mechanisms are used in the two cell types to promote activation of similar downstream effectors. Of particular interest are the roles of the Rho GTPases which control cell morphology and motility via regulation of the actin cytoskeleton. Of particular interest is the role of RhoA, as this GTPase has been described as a master regulator of dendrite morphogenesis [27]. Neurite outgrowth from PC12 cells in response to basic fibroblast growth factor (bFGF) and nerve growth factor (NGF) arises due to p190RhoGAP and ARAP3-mediated inhibition of RhoA [28,29]. Furthermore, activation of RhoA has been shown to inhibit NGF-induced neuronal extension and to promote neurite retraction in PC12 cells. In this study, NGF was shown to inhibit RhoA activity via PKA-mediated phosphorylation on Ser^188^, a process which is required for NGF-induced neurite extension in PC12 cells [30].

Here, we demonstrate that selective activation of PKA entirely mimics the effect of cAMP elevation during initial changes in LNCaP morphology via a process independent of de novo protein synthesis. Furthermore, the effects of cAMP elevation on LNCaP cell morphology can be almost entirely recapitulated following inhibition of RhoA signalling and that RhoA itself is a PKA substrate in these cells. These results suggest that PKA-mediated inhibition of RhoA plays a key role in determining cellular morphology during initial LNCaP cell differentiation to a NE phenotype.

## 2 Materials and methods

### 2.1 Materials

LNCaP cells were purchased from the American Type Culture Collection (ATCC). pRK plasmids containing wild-type and constitutively active myc epitope-tagged RhoA were kind gifts from Professor Alan Hall (Memorial Sloan-Kettering Cancer Centre, New York) whilst Ser188Ala-mutated myc-tagged RhoA was generously donated by Professor Keith Burridge (University of North Carolina School of Medicine, North Carolina). Rhodamine-conjugated phalloidin and the Lipofectamine 2000 transfection reagent were purchased from Invitrogen, Paisley, UK. Myristoylated PKA inhibitor 14-22 amide (myrPKI_14-22_), H89, Y27632, N^6^-benzoyl-cAMP (6-Bnz-cAMP), rabbit polyclonal antibody to pSer^188^RhoA and forskolin (Fsk) were procured from Merck Chemicals Ltd., Nottingham, UK. Cell-permeable C3 transferase (C3T) was purchased from Universal Biologicals Ltd., Cambridge, UK. Complete protease inhibitor cocktail tablets were purchased from Roche Applied Science, Welwyn, UK. Antibodies to RhoA, ERK1/2 and phosphorylated PKA substrate were purchased from Cell Signalling Technology, Hitchin, UK. Antibodies to phospho-Ser^133^ (pSer^133^) CREB were purchased from Abcam, Cambridge, UK. Antibody to receptor of activated C kinase 1 (RACK1) (IgM clone) was purchased from BD Transduction Laboratories, Oxford, UK. Enhanced chemiluminescence reagent and ^3^H-leucine (specific activity 1.48–2.22 TBq/mmol) were purchased from Perkin-Elmer Life Sciences, Waltham, MA. All other reagents were purchased from Sigma-Aldrich, Poole, UK.

### 2.2 Cell culture

LNCaP cells were cultured in a humidified atmosphere at 37 °C, 5% (v/v) CO_2_. Cells were cultured in RPMI1640 medium supplemented with 10%(v/v) foetal bovine serum, 100 U/ml penicillin, 100 μM streptomycin, 1 mM l-glutamine and 1 mM sodium pyruvate. In order to aid cell adherence, all plasticware was coated with 0.1 mg/ml poly-d-lysine HBr prior to use.

### 2.3 Cell transfection

LNCaP cells were seeded into 6 cm tissue culture dishes and allowed to grow to 40–50% confluence. On the day of transfection, growth medium was replaced with RPMI 1640 medium supplemented as described above but lacking antibiotics. Plasmid preparations were defrosted on ice and transfections performed using Lipofectamine 2000 (Invitrogen) as per manufacturer's instructions. The optimal ratio of DNA to Lipofectamine 2000 was found to be 1 μg DNA to 4 μl Lipofectamine 2000. Transfected cells were incubated overnight and cell medium replaced with fresh antibiotic-free growth medium. Cells were then incubated for a further 24 h prior to use in experiments.

### 2.4 Cell stimulation

LNCaP cells were seeded into 6-well tissue culture dishes and grown to 70% confluence prior to experiments. Where appropriate, cells were pre-incubated with the appropriate inhibitor as specified in individual experiments prior to stimulation with 10 μM Fsk. Cell stimulation was quenched by washing cells three times in ice-cold PBS prior to lysis in radioimmunoprecipitation assay buffer containing 50 mM HEPES (pH 7.5), 150 mM sodium chloride, 1%(v/v) Triton X-100, 0.5%(w/v) sodium deoxycholate, 0.1%(w/v) SDS, 5 mM EDTA (pH 8), 10 mM sodium fluoride, 10 mM sodium phosphate, 2 μg/ml benzamidine, 2 μg/ml soybean trypsin inhibitor, 100 μM PMSF and complete protease inhibitor cocktail. Following the addition of 75–100 μl RIPA buffer, samples were solubilised for 30 min on ice. Samples were stored at –80 °C until determination of protein concentration and subsequent immunoblotting. Determination of protein concentration and immunoblotting was performed as described previously [31].

### 2.5 Microscopy and measurement of dendrite outgrowth

Phase contrast microscopy was performed using a Zeiss Axiovert 40 CFL microscope with an AxioCam MRc5 camera attachment. Five random fields per treatment were captured and analysed using Image J software. Dendrite outgrowth was determined by measuring the greatest distance between the cell body and the tip of the extended dendrite. Thirty cells per field were analysed at random and each experiment was repeated three times.

### 2.6 Membrane translocation of RhoA

Cellular membranes from LNCaP cells were isolated as described by Thibault et al. [32]. Briefly, LNCaP cells were seeded into 10 cm tissue-culture dishes and grown to 60–70% confluence. Upon the day of experiment, culture medium was replaced with 5 ml/dish of fresh, supplemented RPMI1640 medium and stimulated as described for individual experiments. Following stimulation, cells were washed 3 times in 5 ml/dish ice-cold PBS and harvested into 300 μl of ice-cold PBS. Cells were pelleted via centrifugation at 5000 *g* for 5 min at 4 °C and subsequently resuspended in 500 μl of ice-cold KCl relaxation buffer containing 100 mM KCl, 50 mM HEPES pH 7.2, 5 mM NaCl, 1 mM MgCl_2_, 0.5 mM EGTA, 100 μM PMSF, 2 μg/ml benzamidine, 2 μg/ml soybean trypsin inhibitor and a complete protease inhibitor. Lysates were sonicated for 2 × 20 s on ice prior to the removal of unbroken cells and nuclei via centrifugation at 700 *g* for 7 min at 4 °C. The resultant supernatant was transferred to a 13 × 51 mm Ultra-Clear™ centrifuge tube and volumes were adjusted to 5 ml in KCl relaxation buffer. Cell membranes were harvested by subsequent ultracentrifugation at 50,000 *g* for 45 min at 4 °C. The supernatant was discarded and the resultant pellet washed in 5 ml of KCl relaxation buffer as described. The washed cell pellet was resuspended in 100 μl of RhoA translocation buffer containing 0.25 M Na_2_HPO_4_, 0.3 M NaCl, 2.5% (w/v) SDS, 100 μM PMSF, 2 μg/ml benzamidine, 2 μg/ml soybean trypsin inhibitor and a complete protease inhibitor. To ensure sufficient solubilisation of the cellular membranes, the lysates were incubated on a rotating wheel at room temperature prior to determination of protein content and fractionation by SDS-PAGE.

### 2.7 Statistical analysis

All statistical analyses were performed using Prism 4 software (GraphPad, San Diego, CA). Data were tested for normality using the Kolomogorov–Smirnov test prior to analysis via either one-way ANOVA with Bonferroni's correction or Kruskal–Wallis test with Dunn's correction.

## 3 Results

**Fig. 1:**
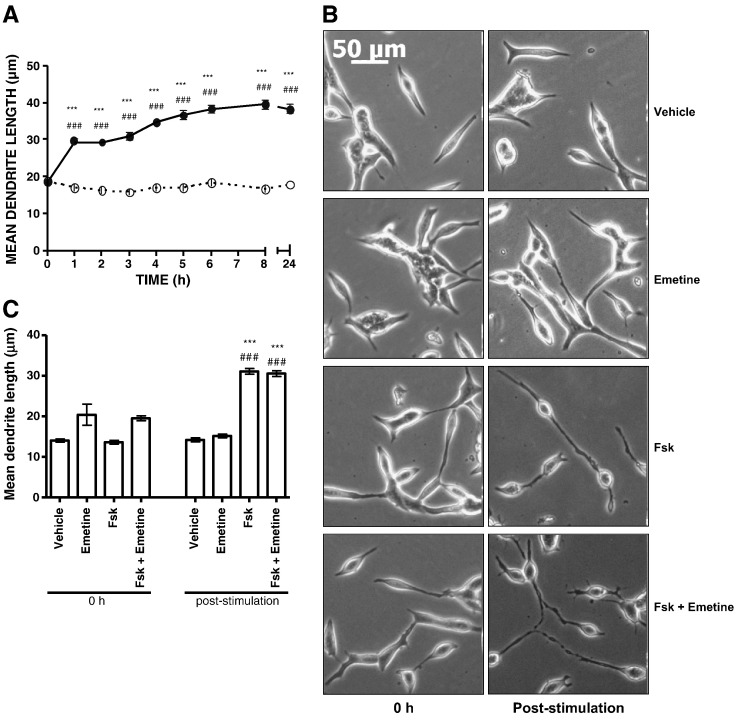
The ability of Fsk to induce rapid changes in LNCaP cell morphology is independent of de novo protein synthesis. Panel A: LNCaP cells were pre-incubated for the indicated times in the presence of either vehicle (0.1% (v/v) EtOH) (open circles) or 100 μM Fsk (closed circles). Mean dendrite outgrowth was determined and results are presented as mean values ± SEM for *n* = 3 experiments. ***p < 0.001 vs. 0 h, ###*p* < 0.001 vs. vehicle at same time point. Panel B: LNCaP cells were pre-incubated for 2 h in the presence of either vehicle (0.1% (v/v) DMSO) or 100 μM protein synthesis inhibitor emetine prior to stimulation with vehicle (0.1% (v/v) EtOH) or 10 μM Fsk for a further 1 h. Phase contrast images were captured immediately prior to the experiment (0 h) and following the 1 h stimulation with Fsk (post-stimulation). Panel C: Mean dendrite outgrowth was assessed at 1 h post-stimulation as described previously. Results are presented as mean values ± SEM for *n* = 3 experiments. ***p < 0.001 vs. 0 h, ###*p* < 0.001 vs. vehicle at same time point.

It has been demonstrated previously that over-expression of constitutively active catalytic PKA subunits can induce terminal NE-like differentiation in LNCaP cells [14] and that PKA-differentiated LNCaP cells can promote the anchorage-independent growth of neighbouring cells [24]. However, the role of PKA in the initial response to elevated cAMP levels in LNCaP cells has not been addressed. Time course experiments in LNCaP cells treated with 10 μM Fsk, a diterpene activator of membrane adenylyl cyclases [33], revealed a time-dependent change in cell morphology consistent with NE-like differentiation and which was detectable at 1 h post-stimulation and sustained for at least 24 h (Fig. 1A). As described by others, the change in LNCaP morphology was characterised by dendrite-like cellular protrusions and branching and rounding of the cell body (Fig. 1B) [12,14,24]. These morphological changes were not observed in either the normal prostate epithelial PZ-HPV-7 cell line or in the tumour-derived DU145 prostate epithelial cell line (data not shown), suggesting that they are specific to the LNCaP cell line. Whilst the cellular processes observed in LNCaP cells morphologically resemble dendrites, they cannot be formally classed as such due to the early time point post-stimulation they were observed. However, for the purpose of this study, these cellular processes will be referred to as dendrites. Importantly, the ability of Fsk to induce changes in LNCaP cell morphology did not require de novo protein synthesis, as pre-treatment with protein synthesis inhibitor emetine at a maximally effective concentration (as determined by inhibition of ^3^H-leucine incorporation into cellular protein) did not significantly alter the observed changes in cell morphology (Fig. 1B,C).

**Fig. 2:**
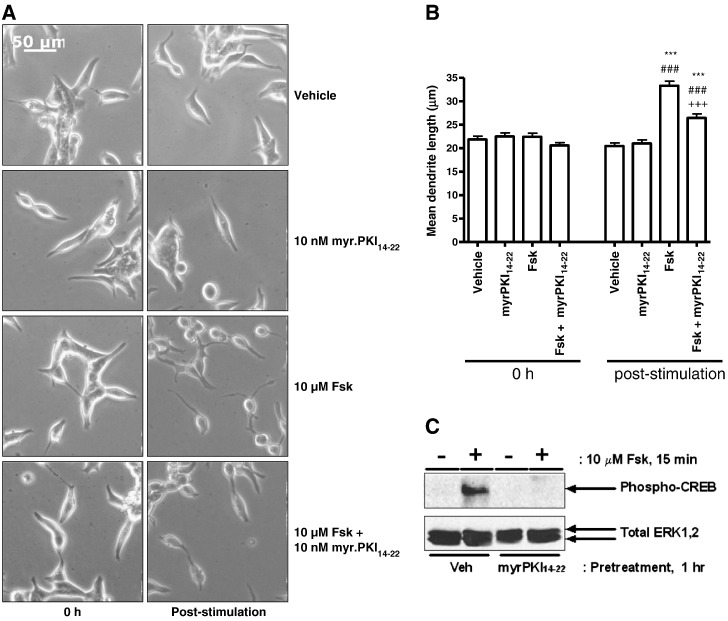
The ability of Fsk to induce changes in LNCaP cell morphology is PKA dependent. Panel A: LNCaP cells were incubated for 1 h in the presence of either vehicle (0.1% (v/v) DMSO) or 10 nM PKA inhibitor myr.PKI_14-22_ prior to stimulation with vehicle (0.1% (v/v) EtOH) or 10 μM Fsk. Phase contrast images were captured immediately prior to the experiment (0 h) and following the 1 h stimulation with Fsk (post-stimulation). Panel B: Mean dendrite length was determined as the distance between the cell body and the tip of the longest dendrite for 30 cells in five random fields for each treatment at each time point. Results are presented as mean values ± SEM for *n* = 3 separate experiments. ***p < 0.001 vs. 0 h, ###*p* < 0.001 vs. vehicle at same time point, +++*p* < 0.001 vs. Fsk at 1 h. Panel C: Inhibitor efficacy was assessed by including control cells which were incubated in the presence and absence of myr.PKI_14–22_ as described above but stimulated for 15 min in the presence of vehicle or 10 μM Fsk to induce phosphorylation of CREB on Ser133. Whole cell lysates were equalised for protein content and volume prior to fractionation via SDS-PAGE and subsequent immunoblotting for pSer^133^CREB as a downstream indicator of PKA activity. Equal protein loading was assessed by determining total ERK1,2 levels.

In order to assess the role of PKA in this phenomenon, LNCaP cells were pre-incubated with the highly PKA-selective cell-permeable peptide inhibitor myr.PKI_14–22_ [16] prior to stimulation with Fsk. Treatment with Fsk induced an increase in mean dendrite length from 22.41 ± 0.78 μm to 33.28 ± 0.99 μm post-stimulation (***p < 0.001 vs. 0 h, ###*p* < 0.001 vs. vehicle; Fig. 2A,B) which was significantly inhibited by pre-incubation with myr.PKI_14–22_ (mean dendrite length post-stimulation = 26.44 ± 0.86 μm, Fig. 1, *p* < 0.001 vs. Fsk-stimulation alone; Fig. 2A, B) at a concentration at which it was effective in blocking PKA-mediated Ser 133 phosphorylation of CREB (Fig. 2C). Incubation with either myr.PKI_14-22_ alone or vehicle failed to induce any change in cellular morphology (Fig. 2A, B).

**Fig. 3:**
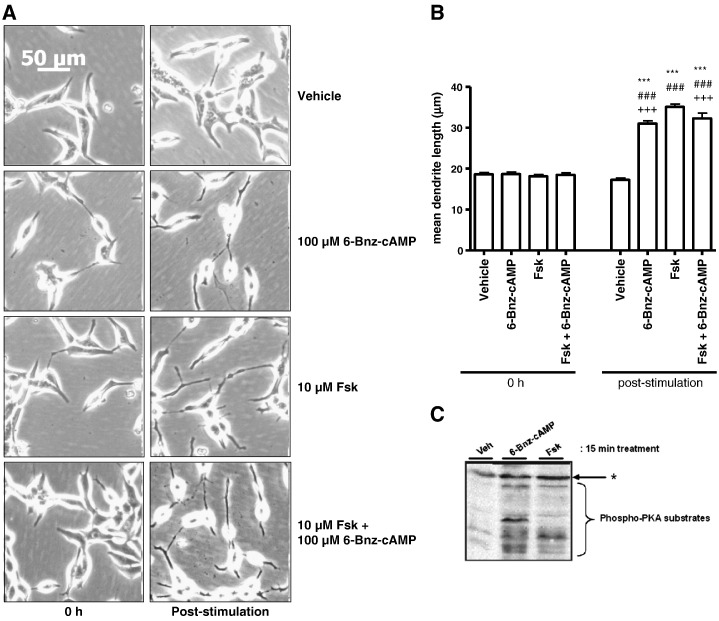
Selective activation of PKA recapitulates the effects of Fsk on LNCaP cell morphology. Panel A: Phase contrast images of LNCaP cells were captured at 0 h and 1 h post-stimulation with vehicle (0.1% (v/v) EtOH, 1% (v/v) DMSO), 10 μM Fsk and/or 100 μM PKA-selective cAMP analogue 6-Bnz-cAMP. Panel B: Changes in mean dendrite length were assessed as described above. Results are presented as mean values ± SEM for *n* = 3 separate experiments. ***p < 0.001 vs. 0 h, ###*p* < 0.001 vs. vehicle at same time point, +++*p* < 0.001 vs. Fsk at 1 h. Panel C: The efficacy of 6-Bnz-cAMP and Fsk was demonstrated in cells stimulated for 15 min. Whole cell lysates were subsequently prepared, equalised for protein content and fractionated via SDS-PAGE and activation of PKA determined by immunoblotting with an anti-phospho-PKA substrate antibody. * indicates a non-specifically labelled immunoreactive band that conveniently serves as a control for equal protein loading.

Whilst the above results indicate that PKA activation is necessary for the NE-like differentiation of LNCaP cells, it does not indicate whether this process is sufficient to induce changes in cell morphology at physiological levels of PKA expression. To address this issue, LNCaP cells were treated with the PKA-selective activator 6-Bnz-cAMP [34] in the presence and absence of Fsk. Treatment with 100 μM 6-Bnz-cAMP entirely recapitulated the effects of Fsk treatment on LNCaP cell morphology with mean dendrite length increasing from 18.68 ± 0.49 to 31.03 ± 0.65 μm (*p* < 0.001 vs. 0 h and vehicle) and from 18.14 ± 0.45 μm to 35.11 ± 0.71 μm (*p* < 0.001 vs. 0 h and vehicle) following stimulation with 6-Bnz-cAMP and Fsk respectively (Fig. 3A, B). The efficacy of 6-Bnz-cAMP and Fsk in activating PKA was confirmed using anti-phospho-PKA substrate antibody, which demonstrated that both stimuli increased PKA-mediated phosphorylation in extracts from treated cells (Fig. 3C) The lack of additivity between 6-Bnz-cAMP and Fsk in mediating NE-like differentiation, coupled with the lack of any significant effect of EPAC activator 007 at concentrations capable of inducing EPAC-regulated gene SOCS-3 [31] (data not shown), indicate that PKA is the dominant effector in this process, in contrast to the synergistic interactions observed between PKA and EPACs in other systems [34].

**Fig. 4:**
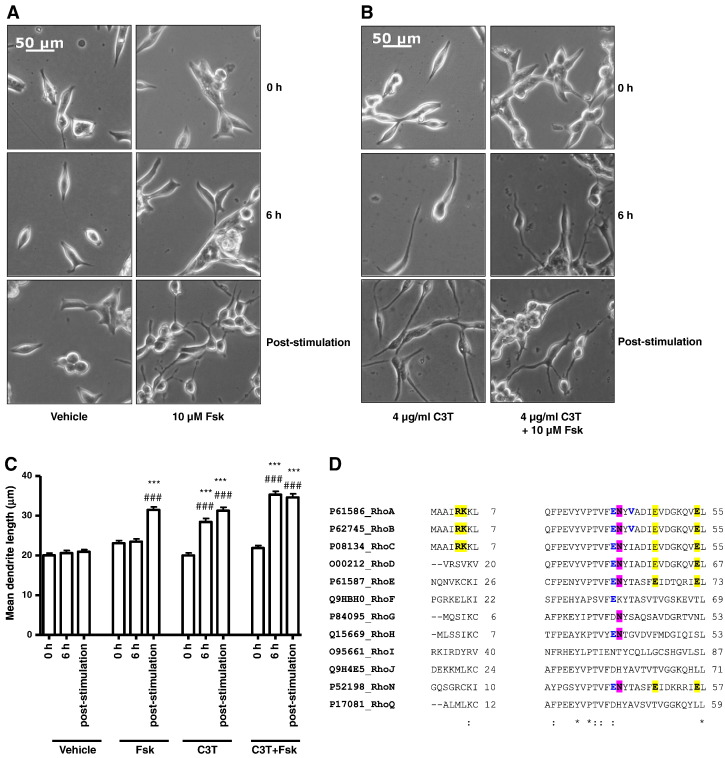
Blockade of Rho activation mimics the effects of PKA. LNCaP cells were plated into 6-well plates and incubated for 6 h with either vehicle (2% (v/v) PBS) (Panel A) or 4 μg/ml C3T (Panel B) prior to stimulation with either vehicle (0.1% (v/v) EtOH) or 10 μM Fsk for 1 h. Images were captured as described at 0 h (0 h), 6 h post-stimulation (6 h) and following stimulation with Fsk (post-stimulation). Panel C: Changes in LNCaP morphology consistent with NE-like differentiation were assessed via increases in mean dendrite length. Results are represented as mean values ± SEM for *n* = 3 separate experiments. ***p < 0.001 vs. 0 h, ###*p* < 0.001 vs. vehicle. Panel D: Protein sequences corresponding to the known human Rho family members retrieved from the UniProt knowledge base were aligned using ClustalW (http://www.clustal.org/). The N-terminus of RhoA was then compared to other family members in order to assess whether they showed similar motifs to those implicated in the binding of C3T to RhoA and subsequent ADP ribosylation (47).  = site of ADP-ribosylation, equivalent to N41  = residues involved in C3T recognition, equivalent to R5, K6, E47 and E54  = residues involved in correct ternary complex formation between Rho, C3T and NAD+, corresponding to E40 and V43. All amino acid positions refer to the position of these residues in RhoA.

Activated PKA exerts its effects via Ser/Thr phosphorylation of downstream targets, thereby altering their activity and/or subcellular localisation [20,35–37]. Interestingly, one reported downstream substrate of PKA is RhoA, a member of the Rho family of small GTPases [38]. PKA-mediated phosphorylation of Ser^188^ inactivates RhoA by preventing dissociation of inactive GDP-bound RhoA from Rho-GDP-dissociation inhibitor (Rho–GDI), thus trapping RhoA in an inactive state [39]. RhoA plays an important role in cellular adherence and morphology by promoting the formation of stress fibres arising from the bundling of actin microfilaments which form the cell cytoskeleton [40,41]. Disruption of the actin polymerisation with cytochalasin B mimicked the effects of Fsk on changes in cell morphology with no evidence of additive interactions (data not shown), suggesting that Fsk is acting to disrupt actin organisation in LNCaP cells. To determine whether these changes in cell morphology were due to inhibition of Rho GTPase-mediated signalling, LNCaP cells were pre-treated with a cell-permeable *Clostridium botulinum* C3 transferase (C3T) for 6 h prior to stimulation with Fsk. C3T inhibits RhoA activation via ADP-ribosylation of Asn41 and is selective for RhoA, RhoB and RhoC members of the Rho family [42]. Treatment of cells with C3T in the absence of Fsk was sufficient to produce a significant increase in mean dendrite length from 24.00 ± 0.79 μm to 33.76 ± 1.07 μm (Fig. 4A–C, *p* < 0.001 vs. vehicle and 0 h). The increase in mean dendrite length observed with C3T alone was comparable to that seen in cells treated with Fsk alone with mean dendrite length increasing from 27.38 ± 0.79 μm at 0 h to 32.85 ± 0.92 μm post-stimulation (*p* < 0.001 vs. 0 h and vehicle). Combined incubation of LNCaP cells with C3T for 6 h followed by treatment with Fsk for 1 h failed to induce a greater increase in mean dendrite length than that observed with Fsk or C3T alone (Fig. 4A–C, mean dendrite length = 24.29 ± 0.84 μm and 36.42 ± 1.16 μm at 0 h and post-stimulation respectively (*p* < 0.001 vs. 0 h and vehicle)). The lack of any additivity between C3T and Fsk treatments suggests that the two are converging on a common mechanism responsible for the observed changes in LNCaP cell morphology. Of the known Rho family members, only RhoA, RhoB and RhoC possess the correct configuration of amino acids to bind and become ADP-ribosylated by C3T (Fig. 4D), thus the effects observed are unlikely to arise from effects on other Rho family members such as RhoG which has also been implicated in neuronal differentiation [43]. However, these observations in isolation do not demonstrate that RhoA itself is essential in this process.

**Fig. 5:**
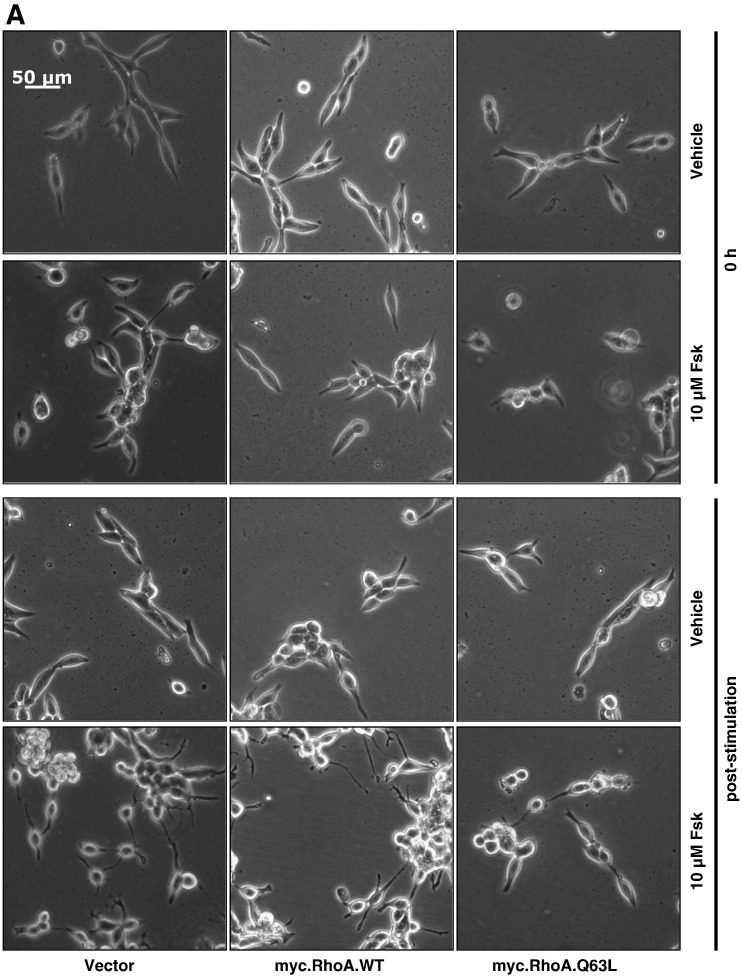

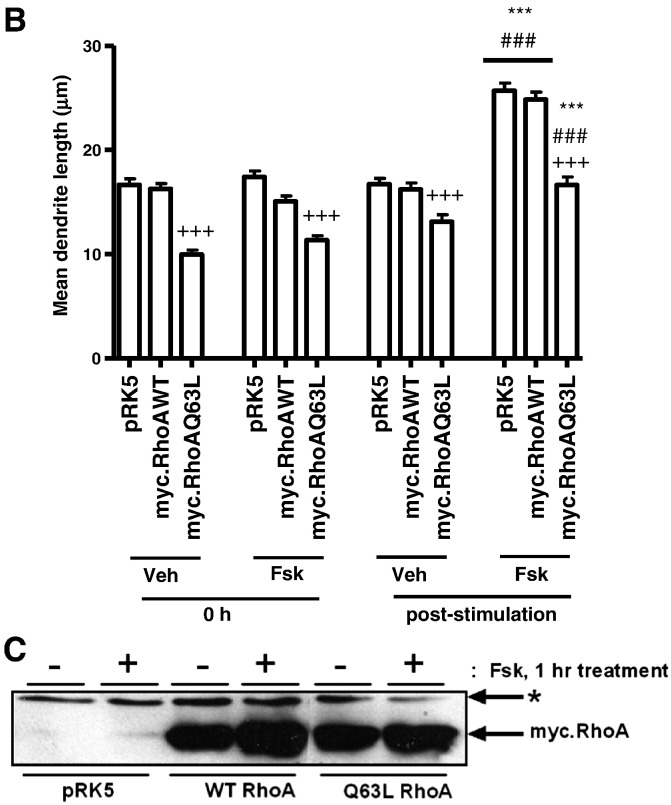
Expression of constitutively active RhoA inhibits Fsk-induced changes in LNCaP cell morphology in a dominant-negative manner. Panel A: LNCaP cells were plated into 6-well plates at a density of 3 × 10^5^ cells per well and transfected with 1 μg of either pRK5, myc.WTRhoA or constitutively active myc.Q63LRhoA as described above. Cells were then stimulated with vehicle (0.1% (v/v) EtOH) and 10 μM Fsk and images captured as described at 0 h (0 h) and 1 h post-stimulation. Panel B: Changes in LNCaP morphology consistent with NE-like differentiation were assessed via increases in mean dendrite length. Results are represented as mean values ± SEM for *n* = 3 separate experiments. ***p < 0.001 vs. 0 h, ###*p* < 0.001 vs. vehicle, +++*p* < 0.001 vs. pRK5. Panel C: Expression of RhoA mutants was assessed via 9E10 immunoblotting for the myc epitope. * indicates a non-specifically labelled immunoreactive band that serves as a control for equal protein loading.

To address this, LNCaP cells were transfected with either vector or a myc-tagged wild-type RhoA (myc.RhoA.WT) or constitutively active GTPase-deficient mutant (myc.RhoA.Q63L) prior to treatment with Fsk. Immediately prior to transfection, all cells appeared identical, whilst at 48 h post-transfection it was noticed that cells expressing myc.RhoA.Q63L displayed shorter cell processes (Fig. 5A, B). Furthermore, expression of constitutively active RhoA significantly blocked the ability of Fsk to induce dendrite-like outgrowth (Fig. 5A, B, *p* < 0.001 vs. Fsk), suggesting that RhoA activation inhibits Fsk-mediated changes in LNCaP cell morphology. This was not simply an effect of RhoA over-expression as cells transfected with myc.RhoA.WT demonstrated changes in cell morphology consistent with that seen in vector-transfected cells (Fig. 5A, B). Moreover immunoblotting with anti-myc tag antibody demonstrated that both WT and Q63L RhoA were expressed at similar levels (Fig. 5C). These results demonstrate that activated RhoA can inhibit the ability of Fsk to induce changes in LNCaP cellular morphology associated with NE differentiation, suggesting that RhoA is the primary Rho GTPase involved in this phenomenon.

**Fig. 6:**
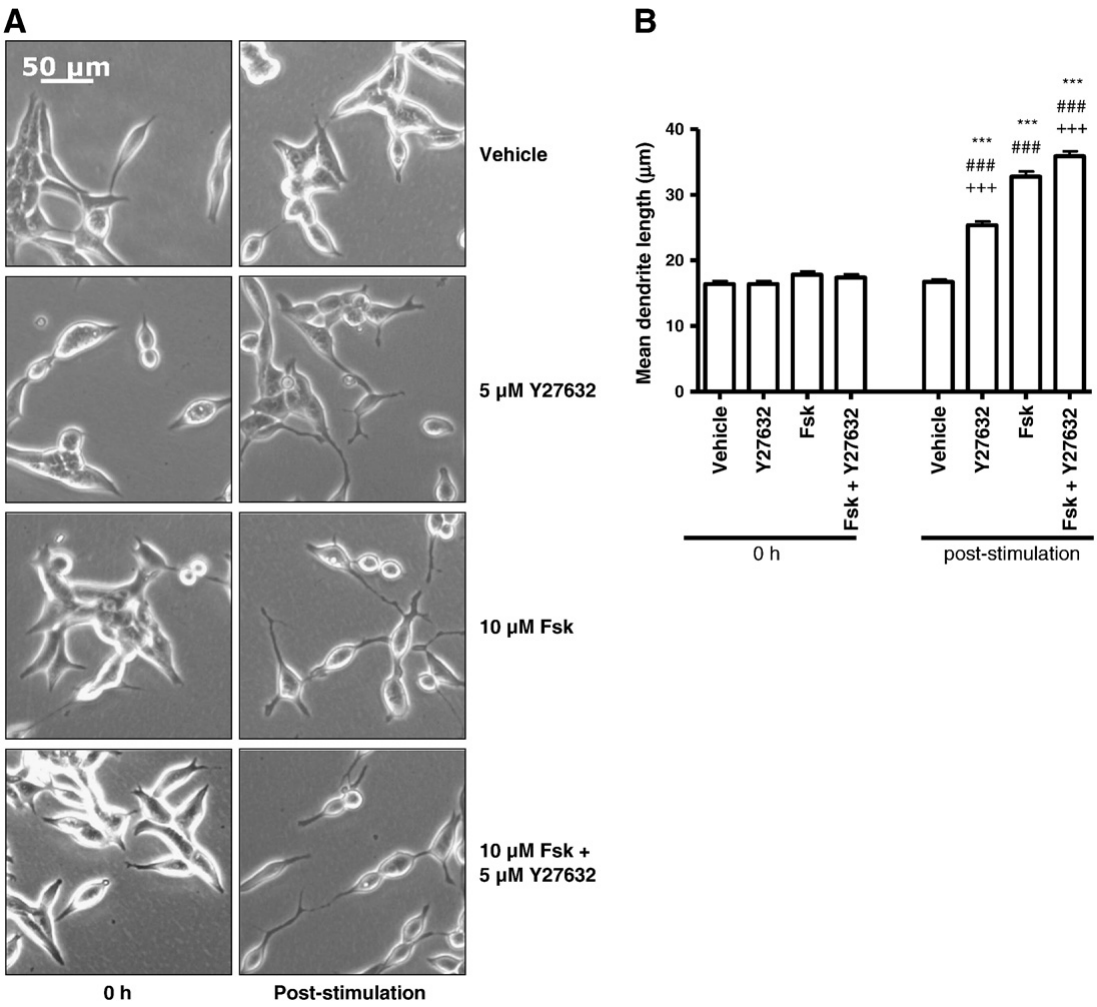
Pharmacological inhibition of ROCK mimics the effects of PKA. Panel A: LNCaP cells were incubated with either vehicle (0.5% (v/v) DMSO) or 5 μM of the ROCK-selective inhibitor Y27632 for 1 h prior to stimulation with either vehicle (0.1% (v/v) EtOH) or 10 μM Fsk for 1 h. Phase contrast images of LNCaP cells were captured at 0 h and immediately following stimulation. Panel B: Mean dendrite length determined (panel B). Results are presented as mean values ± SEM for *n* = 3 separate experiments. ***p < 0.001 vs. 0 h, ###*p* < 0.001 vs. vehicle at same time point, +++*p* < 0.001 vs. Fsk at 1 h.

In neuronal cells, it has been suggested that PKA-mediated inhibition of RhoA activation can promote changes in cellular morphology via a decreased association with the downstream effector Rho-associated protein kinase (ROCK) II [44]. However, unlike previous cell models which derive from the neural crest, LNCaP cells are thought to originate from epithelial stem cells due to their expression of epithelial markers such as PSA [10]. To our knowledge, the role of RhoA in the differentiation of such a cell type has not been previously investigated. To further demonstrate that RhoA activation is important in initial Fsk-induced changes in LNCaP cell morphology, cells were incubated with the ROCK-selective inhibitor Y27632 [45] prior to stimulation with Fsk. As expected, treatment with Y27632 alone promoted an increase in mean dendrite length (Fig. 6A, B, *p* < 0.001 vs. vehicle and Fsk) which was less than that seen with Fsk treatment alone. However, the lack of additivity between Fsk and Y27632 (Fig. 6A, B) suggests that the two pathways exert their effects via a common pathway. These results serve to confirm that inhibition of RhoA-mediated signalling plays a pivotal role in PKA-induced changes in LNCaP cell morphology and that the Rho/ROCK signalling pathway is involved downstream of RhoA.

**Fig. 7:**
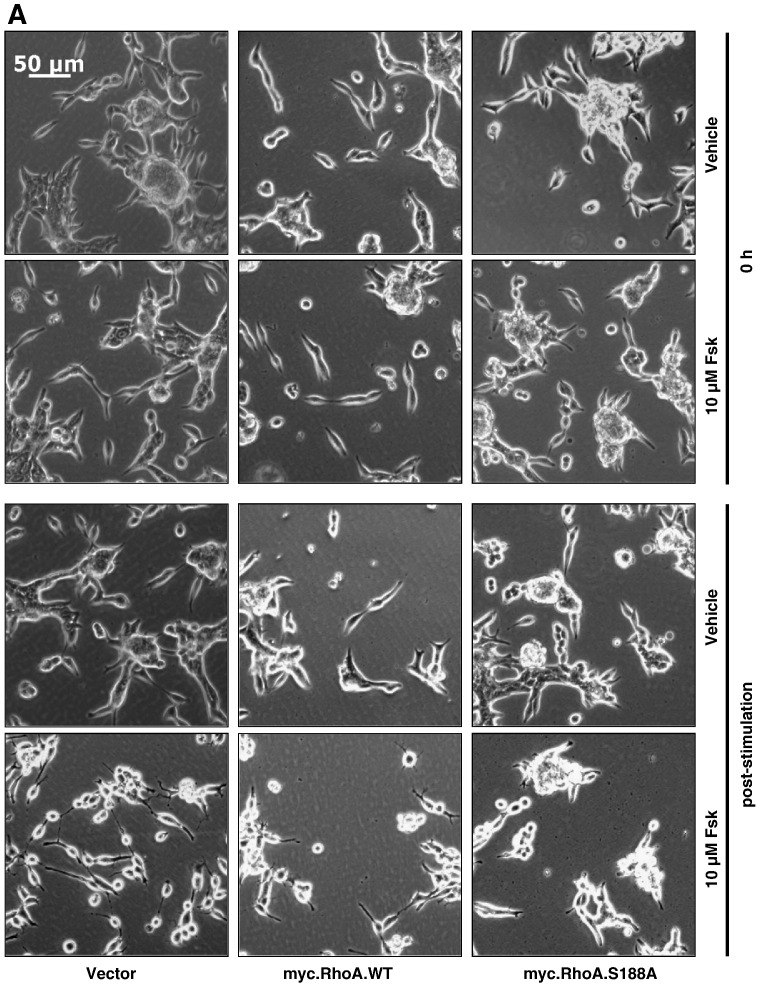

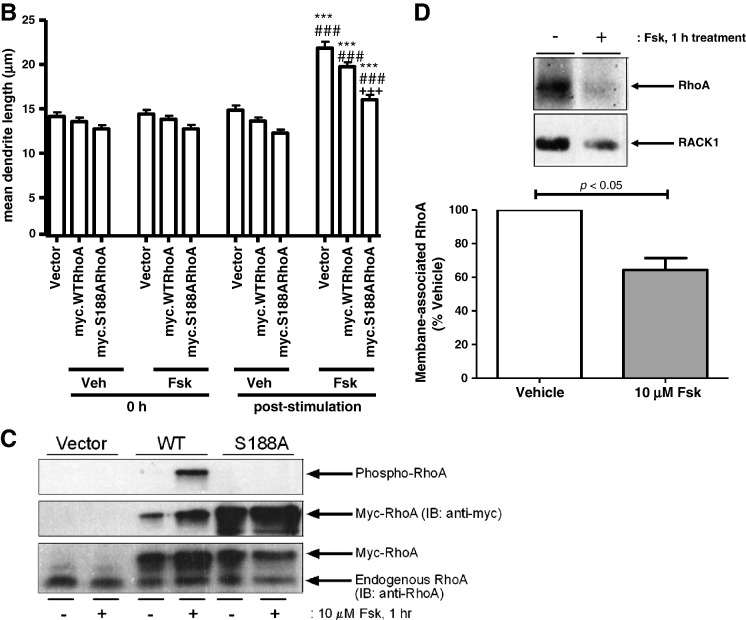
Expression of Ser188Ala-mutated RhoA blocks Fsk-induced changes in LNCaP cell morphology in a dominant-negative manner. Panel A: LNCaP cells were plated into 6-well plates at a density of 3 × 10^5^ cells per well and transfected with 0.5 μg of either pRK5, myc.WTRhoA or myc.S188ARhoA as described above. Cells were then stimulated with vehicle (0.1% (v/v) EtOH) and 10 μM Fsk and images captured as described at 0 h (0 h) and 1 h post-stimulation. Panel B: Changes in LNCaP morphology consistent with NE-like differentiation were assessed via increases in mean dendrite length. Results are represented as mean values ± SEM for *n* = 3 separate experiments. ***p < 0.001 vs. 0 h, ###*p* < 0.001 vs. vehicle, +++*p* < 0.001 vs. vector. Panel C: Expression and changes in RhoA phosphorylation on Ser188 were assessed via immunoblotting for pSer^188^RhoA and total RhoA after treatment with or without 10 μM Fsk for 1 h. Both endogenous and myc-tagged recombinant RhoA expression were detectable by immunoblotting with anti-RhoA antibody. Panel D: Membrane localisation of endogenous RhoA after treatment with or without 10 μM Fsk for 1 h was assessed by immunoblotting of membrane fractions with anti-RhoA antibody. Equalisation for membrane protein loading was assessed using anti-RACK1 antibody. Results are presented as mean values ± SEM for *n* = 3 separate experiments.

It has been demonstrated that PKA can phosphorylate RhoA at Ser188 in vitro and in vivo [38], resulting in the inactivation of downstream RhoA signalling [39,46–48]. To determine whether cAMP elevation promoted serine phosphorylation of RhoA in LNCaP cells and whether this was an important mechanism in mediating the changes in cell morphology observed, LNCaP cells were transfected with vector, myc-tagged wild-type RhoA and a myc-tagged S188A mutant of RhoA prior to stimulation with vehicle or Fsk for 1 h. Whilst all cells displayed similar morphology prior to stimulation, expression of myc.RhoASer188Ala in these cells significantly inhibited Fsk-stimulated increases in mean dendrite length, indicating that phosphorylation of Ser188 of RhoA is necessary for the Fsk-induced changes in LNCaP cell morphology (Fig. 7A, B). Immunoblotting of cell extracts from transfected LNCaP cells revealed that Fsk treatment was able to stimulate phosphorylation of Ser188 in WT RhoA but not the Ser188Ala mutant despite both being expressed at similar levels (Fig. 7C). Consistent with observations that Ser188 phosphorylation inhibits RhoA activity and reduces levels of RhoA at the membrane [49], we found that Fsk treatment resulted in a significant decrease in levels of membrane-associated RhoA (Fig. 7D). Since membrane association of RhoA is associated with its activation, our data suggest that cAMP elevation can promote rapid changes in cell morphology via PKA-mediated phosphorylation and subsequent inactivation of RhoA.

## 4 Discussion

Upon differentiation to a NE phenotype, LNCaP cells display rounding of the cell body and extension of dendrite-like cellular processes. We found that, upon stimulation with the cAMP-elevating compound Fsk, LNCaP cells underwent rapid changes in cellular morphology which could be maximally observed within 1 h of stimulation and occurred independently of de novo protein synthesis. Furthermore, pre-treatment of cells with the PKA-selective inhibitor myr.PKI_14–22_ [16] inhibited Fsk-induced changes in cellular morphology. Similarly, selective activation of PKA with 6-Bnz-cAMP entirely recapitulated the effects of Fsk and the lack of synergistic or additive interactions between Fsk and 6-Bnz-cAMP observed suggests, in accordance with previous data regarding terminal LNCaP NE-like differentiation [14,24], that PKA is the major cAMP sensor involved in the early morphological changes observed in LNCaP cells. Such results are in keeping with observations that over-expression of a constitutively active mutant of the catalytic subunits of PKA in LNCaP cells promotes NE differentiation in the absence of other stimuli and furthermore enhances both the anchorage-dependent and -independent growth of PCa cells in vitro and in vivo [14,24]. However, over-expression models are not ideal as high levels of protein expression may not be physiologically relevant and the focus of those studies was terminally differentiated cells. To our knowledge, this is the first demonstration of a central role of PKA in the early morphological changes in LNCaP cell differentiation towards a NE-like phenotype. It should be noted here that our assessment of differentiation is based solely on morphological changes. Gene and protein markers of LNCaP differentiation that have been employed include neuron-specific enolase and chromogranin A [12,15]. However undifferentiated cells express detectable levels of both proteins, and the small fold-changes in expression that we (data not shown) and others have observed even over sustained differentiation time courses limit their utility as differentiation markers, particularly in examining early changes that occur independently from new protein synthesis.

Whilst PKA can phosphorylate transcription factors such as CREB [18–20], it is unlikely that the rapid effects seen are due to de novo gene transcription as the rapid change in cell morphology observed was coupled with an inability of pre-treatment with emetine to block Fsk-mediated changes in LNCaP cell morphology under conditions in which it completely inhibited new protein synthesis. This would suggest that the primary mechanism governing changes in cell morphology is PKA-mediated phosphorylation of downstream effectors. One potential candidate was the small GTPase RhoA which plays an important role in regulating actin cytoskeletal dynamics. It has been demonstrated previously that PKA can directly phosphorylate RhoA in vitro on Ser188 [38], which forms part of a consensus motif for PKA-mediated phosphorylation (K^185^KKSG) [49]. Furthermore, substitution of this residue to a non-phosphorylatable Ala residue inhibited cAMP-mediated changes in NE morphology [30,44]. Moreover, inhibition of ROCK, a downstream effector of RhoA, can also promote changes in cellular morphology consistent with NE-like differentiation in LNCaP cells [44]. In our study, it was found that treatment of LNCaP cells with either C3T to inhibit RhoA or blockade of ROCK signalling using Y27632 promoted changes in cellular morphology comparable to that seen with Fsk treatment alone. In accordance with these observations, over-expression of a constitutively active mutant of RhoA inhibited dendrite-like extensions in both the presence and absence of Fsk. Attempts to over-express a dominant negative mutant of RhoA were unsuccessful, due to decreased cellular adherence to the substratum which was particularly problematic in LNCaP cells which have previously been reported to display poor adherence even under normal culture conditions [10]. However, taken together, these data would suggest that PKA-mediated inhibition of RhoA signalling plays an important role in cAMP-mediated changes in LNCaP cellular morphology. Furthermore, treatment with Fsk promoted an increase in pSer^188^RhoA which was blocked upon prior incubation with myr.PKI_14–22_ and expression of myc.RhoAS188A inhibited Fsk-induced changes in cellular morphology in a dominant-negative fashion

Taken together these results provide compelling evidence that rapid, early changes in LNCaP cell morphology consistent with NE-like differentiation are governed by PKA-mediated phosphorylation of RhoA on Ser^188^ and subsequent inhibition of downstream signalling. Phosphorylation of RhoA on this residue inhibits its actions via several mechanisms. Firstly, pSer^188^RhoA remains associated with Rho-GDI which locks RhoA in a GDP-bound, inactive state and thus prevents it from cycling back to the active GTP-bound form [49]. Secondly, one of the hallmarks of RhoA activation is its translocation to the cell membrane, however association with Rho-GDI sequesters Ser^188^-phosphorylated RhoA in the cytoplasm [39,48], thus preventing it from exerting any effects at the membrane. Stimulation with Fsk decreased membrane association of RhoA in LNCaP cells, further suggesting inactivation of RhoA. Thirdly, it has been observed that pSer^188^RhoA displays a decreased capacity to bind and activate its downstream effector ROCK [30]. Whilst RhoA has many downstream effectors, the observation that Y27632 can recapitulate the effects of cAMP elevation suggests that RhoA/ROCK signalling plays the primary role in this phenomenon. However, whilst likely to play a central role in early differentiation in LNCaP cells, the PKA/RhoA/ROCK pathway suggested here is by no means exclusive. This is particularly pertinent when considering the observation that overexpression of myc.Ser188AlaRhoA failed to completely block Fsk-induced changes in cell morphology, implying that other signalling pathways are potentially involved in mediating PKA's effects.

Whilst PC12 cells differ from LNCaP cells in that integration of both PKA and EPAC-regulated signals are required for neurite outgrowth, a role for PKA-mediated inhibition of RhoA downstream of the NGF receptor has been described. In this study, phosphorylation of RhoA on Ser^188^ was reported [30] although other pathways have been implicated in NGF-mediated inhibition of RhoA. It has been suggested that both basic fibroblast growth factor- and NGF-mediated neurite outgrowth in these cells is facilitated by p190RhoGAP and ARAP3-mediated inhibition of RhoA [28,29]. It is possible that the relative importance of the different signalling pathways may be temporally regulated. Both studies investigated the role of RhoA in neurite outgrowth in PC12 cells. However, whilst the recent work by Jeon and co-workers investigated signalling pathways activated following NGF and bFGF at several hours post-stimulation [28,29], the work by Nusser and co-workers [30] investigated the impact of NGF on RhoA at 20 and 120 min post-stimulation. It is therefore possible that the immediate changes in cell morphology which we have investigated arise primarily through PKA-mediated phosphorylation of RhoA on Ser^188^ but that p190RhoGAP and ARAP3 may play a role in maintaining these changes at later time points. Our data suggest that PKA-mediated phosphorylation of RhoA on Ser188 and subsequent inhibition of ROCK signalling is the dominant signalling pathway involved in rapid changes in LNCaP cellular morphology.

Whilst much work has focused on terminal differentiation of LNCaP cells to a NE phenotype, there is little published data on pathways involved in the early stages of this process during which therapeutic intervention may be most beneficial. It is possible therefore that selective inhibition of the RhoA/ROCK signalling pathway by manipulation of cAMP-mediated signalling may prove an effective and novel adjuvant to current therapeutic strategies.

## 5 Conclusions

• Elevation of cAMP rapidly initiates the development of a neuroendocrine-like phenotype in LNCaP prostate epithelial cells.
• PKA activation is necessary and sufficient for initiation of the phenotype.
• PKA stimulates phosphorylation of RhoA on Ser188, and expression of Ser188Ala-mutated RhoA blocks PKA-mediated development of the neuroendocrine-like phenotype.
